# Amino acid tracers in PET imaging of diffuse low-grade gliomas: a systematic review of preoperative applications

**DOI:** 10.1007/s00701-018-3563-3

**Published:** 2018-05-24

**Authors:** Olivia Näslund, Anja Smits, Petter Förander, Mats Laesser, Jiri Bartek, Jens Gempt, Ann Liljegren, Eva-Lotte Daxberg, Asgeir Store Jakola

**Affiliations:** 1 Sahlgrenska Academy, Medicinaregatan 3, 41390 Gothenburg, Sweden; 2 Institute of Physiology and Neuroscience, Sahlgrenska Academy, Gothenburg, Sweden; 3 Department of Neuroscience, Neurology, Uppsala University, Uppsala, Sweden; 4 Department of Clinical Neuroscience and Department of Medicine, Karolinska Institutet, Stockholm, Sweden; 5 Department of Neurosurgery, Karolinska University Hospital, Stockholm, Sweden; 6 Department of Radiology, Institute of Clinical Sciences, The Sahlgrenska Academy at University of Gothenburg, Gothenburg, Sweden; 7 Department of Neurosurgery, St. Olavs Hospital, Trondheim, Norway; 8 Department of Neurosurgery, Copenhagen University Hospital Rigshospitalet, Copenhagen, Denmark; 9 Department of Neurosurgery, Klinikum rechts der Isar, Technical University of Munich, Munich, Germany; 10 Department of Radiology, Sahlgrenska University Hospital, Gothenburg, Sweden; 11 Medical Library, Sahlgrenska University Hospital, Gothenburg, Sweden; 12 Department of Neurosurgery, Sahlgrenska University Hospital, Gothenburg, Sweden

**Keywords:** Amino acid, Biopsy, Glioma, PET, Prognosis

## Abstract

Positron emission tomography (PET) imaging using amino acid tracers has in recent years become widely used in the diagnosis and prediction of disease course in diffuse low-grade gliomas (LGG). However, implications of preoperative PET for treatment and prognosis in this patient group have not been systematically studied. The aim of this systematic review was to evaluate the preoperative diagnostic and prognostic value of amino acid PET in suspected diffuse LGG. Medline, Cochrane Library, and Embase databases were systematically searched using keywords “PET,” “low-grade glioma,” and “amino acids tracers” with their respective synonyms. Out of 2137 eligible studies, 28 met the inclusion criteria. Increased amino acid uptake (lesion/brain) was consistently reported among included studies; in 25–92% of subsequently histopathology-verified LGG, in 83–100% of histopathology-verified HGG, and also in some non-neoplastic lesions. No consistent results were found in studies reporting hot spot areas on PET in MRI-suspected LGG. Thus, the diagnostic value of amino acid PET imaging in suspected LGG has proven difficult to interpret, showing clear overlap and inconsistencies among reported results. Similarly, the results regarding the prognostic value of PET in suspected LGG and the correlation between uptake ratios and the molecular tumor status of LGG were conflicting. This systematic review illustrates the difficulties with prognostic studies presenting data on group-level without adjustment for established clinical prognostic factors, leading to a loss of additional prognostic information. We conclude that the prognostic value of PET is limited to analysis of histological subgroups of LGG and is probably strongest when using kinetic analysis of dynamic FET uptake parameters.

**Electronic supplementary material:**

The online version of this article (10.1007/s00701-018-3563-3) contains supplementary material, which is available to authorized users.

## Introduction

Diffuse low-grade glioma (LGG) is a relatively rare brain tumor typically presenting in young adults. The course of disease is variable, but the natural history of LGG includes continuous growth with eventually tumor progression and impaired life expectancy. Inactive lesions may lack clear visible signs of apparent growth for several years, while some LGG experience rapid malignant transformation [9]. Although time to malignant transformation is very heterogeneous at the individual level, one recent study found the incidence of malignant transformation to be 0.17 per person year among LGG patients [31]. To no surprise, malignant transformation is strongly linked to impaired survival [22, 31]. Baseline variables such as age, functional status, and size are used to predict the disease course in individual patients, but also the uptake of amino acids in the tumor measured by PET is reported to be of prognostic value [10, 34].

Magnetic resonance imaging (MRI) is the diagnostic modality of choice when it comes to LGG. Some LGG have contrast enhancement within the tumor area, although no necrosis is seen [49]. In 15–29% of all LGG, focal and patchy contrast enhancement may be present, towards which biopsies are often targeted [8]. Nevertheless, gadolinium-enhanced MRI lacks sensitivity for anaplastic foci [19]. In addition to providing inadequate prognostic information, under-grading has implications with respect to the choice and timing of adjuvant therapy. Hence, imaging modalities such as PET that more accurately reflect underlying tumor biology are supposedly of value to avoid sampling bias.

PET imaging with labeled amino acid tracers has been widely used to capture biological activity of LGG. The most commonly used amino acid tracers in brain tumor imaging are 18F-fluoro-ethyl-l-tyrosine (FET) and 11C-methyl-l-methionine (MET) [40]. In spite of the widespread use of PET in LGG, there is no clear evidence for the clinical benefit in terms of diagnostic and prognostic capabilities for these patients. The objective of this systematic review was to investigate the clinical value of the different preoperative applications of amino acid PET in LGG. Due to the complexity of this topic, the review was subdivided to answer four specific questions:

1. Is PET helpful in differentiating suspected LGG from high-grade gliomas (HGG) and from lesions of non-neoplastic origin?
2. Do increased uptake ratios (lesion/ brain) in areas targeted by PET-guided biopsies correlate to higher malignancy grade of suspected LGG?
3. What is the prognostic information provided by preoperative PET imaging after adjusting for established prognostic variables in LGG?
4. Finally, can preoperative PET with amino acid tracers predict molecular subgroups in LGG?

## Methods

**Fig. 1:**
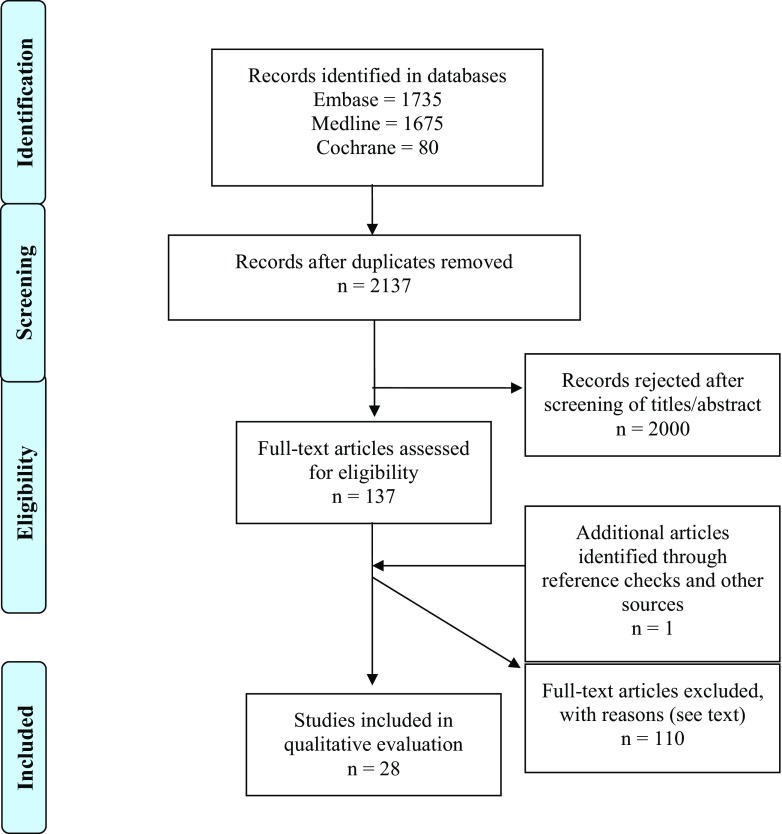
Resulting flow chart of search strategy

A systematic review of the current literature was undertaken searching Medline, Cochrane Library, and Embase. With the help of librarians at the Medical Library at Sahlgrenska University Hospital, a search designed to include keywords “PET,” “low-grade glioma,” and “amino acid tracers” with synonyms was designed. See supplementary results for a complete list of search commands. Both free-text and subject headings were used, with Medical Subject Headings (MeSH) as the standard for PubMed and EMTREE in Embase. Delimitations were publication date before 1995 and publication language other than English, Swedish, Danish, and Norwegian. The most recent search was made January 9, 2017, and any duplicates of articles were removed before librarians submitted the literature retrieval to the authors (Fig. 1).

One author (O.N.) evaluated the conformity of each study with the objectives of the current systematic review. Case reports, meeting abstracts, commentaries, and reviews were excluded, as well as studies involving mixed populations or similar patient cohorts. The studies selected for full-text analysis were divided between the authors and independently analyzed by two authors per study. Any discrepancies were solved by discussion, and if a consensus could not be reached, a third opinion from two senior authors (A.J. or A.S.) gave the final verdict. A standardized form was used to summarize and extract data. Authors were contacted to supplement data if none was available and deemed to be of value.

### Statistics

Only descriptive data are presented without any attempt at pooled estimates/meta-analysis due to the expected considerable heterogeneity of studies in terms of design, tracers, thresholds, and outcomes.

## Results

After removing duplicates, 2137 articles were identified for further screening. Evaluation of titles and abstracts resulted in the removal of 2000 articles, leaving 137 for full-text analysis, of which 28 were deemed suitable for inclusion in this systematic review (Fig. 1 and Table 1). As shown in Table 1, PET studies with different amino acid tracers, mostly 18F-FET (*n* = 9) and 11C-MET (*n* = 6) and using static and dynamic uptake methods, were included. The results of these 28 publications with regard to the four specific questions raised in this review are summarized below.

**Table 1:** Compilation of included study data

Author	Tracer	Uptake	Cutoff value	Published	Type of study	Number of patients	Single center	WHO classification
Albert et al. [2]	FET	Both	TBR_max_ ≥ 2.7	2015	R	314	Yes	2007
Berntsson et al. [4]	MET	Static	N/A	2013	P	24	Yes	2007
Bette et al. [5]	FET	Static	TBR_max_ ≥ 1.3	2016	R	65	Yes	2007
Bisdas et al. [6]	MET	Static	N/A	2013	P	28	Yes	Older
De Witte et al. [10]	MET	Static	N/A	2001	R	85	Yes	Older
Dunet et al. [12]	FET	Dynamic	N/A	2014	P	42	Yes	2007
Ewelt et al. [13]	FET	Static	TBR ≥ 1.6	2011	P	30	Yes	2007
Floeth et al. [14]	FET	Static	TBR ≥ 1.6	2005	P	91	Yes	Older
Floeth et al. [15]	FET	Static	TBR ≥ 1.6	2011	P	25	Yes	2007
Gumprecht et al. [18]	MET	Static	T/N ratio ≥ 1.5	2007	?	20	Yes	Unclear
Herholz et al. [20]	MET	Static	N/A	1998	R	196	Yes	Older
Hutterer et al. [21]	FET	N/A	N/A	2013	R	95	No	Unclear
Jansen et al. [24]	FET	Both	N/A	2012	R	144	Yes	2007
Jansen et al. [25]	FET	Dynamic	SUV/BG ≥ 1.8	2014	R	59	Yes	2007
Jansen et al. [23]	FET	Dynamic	SUVmax ≥ 1.8	2012	R	127	Yes	Unclear
Kunz et al. [27]	FET	Dynamic	N/A	2011	P	55	Yes	2007
Malkowski et al. [30]	N/A	N/A	TBR ≥ 1.6	2015	?	N/A	N/A	N/A
Pauliet et al. [32]	FET	N/A	TBR ≥ 1.6	2005	P	31	Yes	2007
Pichler et al. [33]	FET	Static	N/A	2010	R	88	Yes	Unclear
Pyka et al. [35]	FET	Both	TBR ≥ 1.2	2014	R	34	Yes	2007
Pöpperl et al. [36]	FET	Dynamic	N/A	2007	P	54	Yes	Unclear
Roessler et al. [38]	MET	Static	N/A	2007	P	27	Yes	Unclear
Smits et al. [41]	MET	Static	HS/cortex ratio ≥ 2.1	2008	R	129	Yes	Older
Takano et al. [43]	MET	N/A	T/N ratio ≥ 1.2	2016	R	35	Yes	2007
Thon et al. [44]	FET	Dynamic	SUV/BG ≥ 1.8	2014	P	98	Yes	2007
Unterrainer et al. [45]	FET	Both	N/A	2016	R	31	Yes	2007
Watanabe et al. [47]	MET	N/A	N/A	2015	R	163	Yes	2007
Widhalm et al. [48]	MET	N/A	T/N ratio ≥ 1.5	2010	?	35	Yes	2007

*N/A* not available; *MET* 11C-methionine; *FET* 18F-fluoro-ethyl-tyrosine; *T/N ratio* tumor-to-normal uptake ratio; *TBR*_*max*_ tumor-to-background ratio max; *SUV/BG* standardized uptake values/background; *SUV*_*max*_ standardized uptake values max; *HS/cortex ratio* hot spot-to-cortex ratio; *WHO* World Health Organization; *R* retrospective; *P* prospective

### Amino acid PET to differentiate LGG from other entities

In a population of presumed LGG patients, Jansen et al. [23] noted increased FET uptake in 49/73 tumors that were confirmed as LGG (69%) and in 42/47 tumors classified as HGG (89%), demonstrating that although both tumor entities can harbor increased FET signal, uptake is more often increased in HGG. Pöpperl et al. [36] demonstrated an increased FET uptake in 13/24 (54%) patients with MRI-suspected LGG, of which 9/13 (69%) lesions were histopathological proven LGG. Gumprecht et al. [18] studied 20 patients with presumed LGG and found increased MET uptake in 16 patients (80%). However, the association with histopathology revealed increased MET uptake in 1/4 (25%) confirmed WHO grade II gliomas, 13/14 (93%) of grade III gliomas, and 2/2 (100%) of grade IV gliomas. Similar findings showing increased uptake in both LGG and HGG,but, more frequently in HGG, have been observed in several other studies, as shown in Table 2 [14, 15, 18, 23, 38]. In summary, the uptake of amino acid measured by PET in presumed LGG based on conventional MRI was increased in 25–92% of subsequently histopathological verified LGG and in 83–100% of histopathological verified HGG. In addition, some unspecific findings consisting of non-neoplastic lesions were encountered. Jansen et al. [23] observed increased FET uptake in 5/7 non-neoplastic lesions (57%), while Floeth et al. [14] observed increased FET uptake in 1/10 (10%) lesions that proved to be non-neoplastic (Table 2).

**Table 2:** Studies on amino acid PET in suspected low-grade glioma

Author	Presumed LGG, *n*	Presumed LGG PET+, *n*/*N* (%)	PET+ in non-neoplastic lesions, *n*/*N* (%)	PET+ in confirmed grade 2 gliomas, *n*/*N* (%)	PET+ in confirmed grade 3 gliomas, *n*/*N* (%)	PET+ in confirmed grade 4 gliomas, *n*/*N* (%)
Berntsson et al. [4]	24	18/24 (75)	0/1 (0)	13/18 (72)	5/5 (100)	N/A
Floeth et al. [15]	25	22/25 (88)	N/A	7/16 (44)	14/16(88)	1/1 (100)
Floeth et al. [14]	23	11/23 (48)	1/10 (10)	5/7(71)	5/6 (83)	N/A
Dunet et al. [12]	38	36/38 (95)	N/A	N/A	N/A	N/A
Gumprecht et al. [18]	20	16/20 (80)	N/A	1/4 (25)	13/14 (93)	2/2 (100)
Hutterer et al. [21]	54	37/54 (69)	N/A	N/A	N/A	N/A
Jansen et al. [23]	127	97/127 (76)	4/7 (57)	49/71 (69)	37 /47 (89)	5/5 (100)
Pöpperl et al. [36]	24	16/24 (67)	N/A	13 /22 (grade 2 + 3) (59)	N/A	N/A
Roessler et al. [38]	12	11/12 (92)	N/A	11/12 (92)	8/8 (100)	4/4 (100)
Takano et al. [43]	35	34/35 (97)	N/A	22/23 (96)	12/12	N/A
Thon et al. [44]	133	102/133 (77)	N/A	N/A	N/A	N/A

*N/A* not available

### PET-guided biopsies towards areas of focal increased uptake

Few investigations have explicitly covered PET-guided biopsies in presumed LGG. PET hot spots (i.e., areas with highest tracer uptake) in MRI-suspected LGG were reported in the range of 11–96% [4, 27, 38, 44]. In two studies using static uptake of MET and biopsies targeted at focal hot spots in presumed LGG, one study reported that 3/6 (50%) tumors were grade II, 1/6 (%) was grade III, and 2/6 (%) were grade IV [38], while the other study showed that 17/23 (%) were grade II, 5/23 (%) grade III, and 1/23 (%) non-neoplastic [4]*.* Roessler et al. [38] found a higher percentage of malignant gliomas when using static MET uptake, showing that 8/12 cases (67%) had hot spot area on PET that corresponded to grade III malignancy.

Kunz et al. [27] divided a cohort (*n* = 55) of presumed LGG into three groups based on dynamic FET PET characteristics. In 15 patients, a heterogeneous imaging pattern was present, where areas with steady increasing metabolic activity coexisted together with areas with an early peak of metabolic activity followed by a constant decline. Of these tumors, 1/15 (7%) was classified as grade II glioma while 14/15 tumors (93%) were grade III gliomas.

Thon et al. [44] similarly analyzed three different groups in presumed LGG with respect to contrast uptake kinetics using dynamic FET PET. They found a subgroup of tumors showing focal decreasing time-activity curves (TAC), suggestive of lesions harboring hot spots. Histopathological analysis of this subgroup with focal decreasing TAC revealed that 2/19 (11%) tumors were grade II gliomas and 17/19 (89%) were grade III gliomas. In addition, the study by Kunz et al. demonstrated a clear correlation between hot spot and malignancy grade; from each tumor, several biopsy samples were harvested, and while histopathological evaluation of specimens from inside the hotspot (*n* = 67) revealed WHO grade III glioma in 57 samples (85%), WHO grade II glioma was revealed in 46 samples (90%) derived from areas outside the hotspot (*n* = 51) [27]*.*

### Independent prognostic information provided by preoperative PET imaging

Only two studies [25, 41] have addressed the issue of how amino acid uptake measured by PET performs in prognostication when adjusted for clinically important factors in LGG. While Jansen et al. [25] reported a hazard ratio (HR) of 0.77 (*p* = 0.50) for FET uptake, Smits et al. [41] described a HR of 2.69 (*p* = 0.002) in astrocytomas and HR of 1.29 (*p* = 0.16) in oligodendrogliomas for MET uptake (Table 3). When adjusted for molecular markers, the prognostic capabilities of PET in patients with LGG were described by only a single study [44]. Thon et al. [44] analyzed the risk of increased FET uptake in presumed LGG and found a HR of 1.8 (*p* = 0.003), adjusted for *IDH* status, 1p/19q codeletion, and Karnofsky performance status. To be noted is that this study used kinetic analysis of different TAC in cases with increased FET uptake and thus excluded tumors with normal or decreased uptake ratios on dynamic scans.

**Table 3:** Prognostic studies of amino acid PET

Author	IDH mutation	1p19q codeletion	Prognostic variables available	Risk of PET+, adjusted^a^	Risk of PET+, Molecular adjusted
Berntsson et al. [4]	No	Yes	Yes	No	No
Bette et al. [5]	Yes	Yes	Yes	No	No
Jansen et al. [25]	No	No	Yes	HR 0.77, *p* = 0.50	No
Smits et al. [41]	No	No	Yes	Astrocytomas HR = 2.69, p = 0.002, oligodendrogliomas HR = 1.29, *p* = 0.1561	No
Takano et al. [43]	No	No	Yes	No	No
Thon et al. [44]	Yes	Yes	Yes	No	HR = 1.8, **p = 0.003 All lesions PET+
Unterrainer et al. [45]	No	No	Yes	No	N/A

** Adjusted for IDH 1/2 status

^a^Yes = adjusted for at least two out of three of the following prognostic variables: age, tumor size, or functional status

### Amino acid PET to predict molecular subgroups in LGG

So far, two investigations attempted to predict molecular class based on amino acid PET imaging. These reports by Bette et al. [5] and Thon et al. [44] provided conflicting evidence with respect to PET uptake in the respective molecular groups in newly diagnosed or presumed LGG. Increased FET uptake was reported in 50 versus 100% of *IDH* mutated 1p19q codeleted LGG (i.e., oligodendrogliomas), 32 versus 89% in *IDH* mutated non-codeleted LGG (i.e., astrocytomas), and 66 versus 83% in *IDH* wild-type LGG.

## Discussion

In this systematic review, we found that amino acid uptake ratios measured by PET can be increased in LGG, HGG, and non-neoplastic cerebral lesions. Concerning PET-guided biopsies, dynamic FET imaging seems superior to other reported techniques with respect to detecting corresponding focal areas of higher malignancy. There are limited and conflicting findings with respect to independent prognostic information from PET imaging. Finally, there is no current support for the clinical value of PET with respect to prediction of molecular tumor status of LGG.

### Amino acid PET differentiates LGG from other entities

Our first question concerns the preoperative value of PET in presumed LGG, i.e., the ability of amino acid PET to differentiate LGG from lesional non-neoplastic diagnoses and HGG. We found consistent results of increased PET uptake in both non-neoplastic lesions, LGG, and HGG, although more frequently and higher uptake values in HGG [14, 15, 18, 23, 36, 38]. Based on the inclusion of the selected studies, it is clear that the diagnostic accuracy by conventional MRI is problematic given the high number of HGG in the group of presumed LGG, and this is exemplified by Scott et al. who report 21/243 (9%) malignant gliomas lacking contrast enhancement [39]. Another explanation may be a drift towards more liberal inclusion in the group “presumed LGG” due to researchers wish to include as many patients as possible of this relative rare entity in clinical studies, improving study power but at the cost of more heterogeneous data. Nevertheless, due to the considerable overlap in amino acid uptake values, a clear separation of LGG from HGG by PET seems problematic. This is a well-recognized problem related to the generally higher tracer uptake in oligodendrogliomas compared to astrocytomas, causing overlap between oligodendrogliomas and HGG [28]. For instance, Roessler et al. [38] found no difference in tracer uptake between malignant astrocytomas and low-grade oligodendrogliomas by MET PET. Further, Jansen et al. [23] found higher FET uptake values in HGG than LGG, but no significant differences between the mean values for uptake parameters derived from static FET images between HGG and LGG. However, after exclusion of oligodendroglial tumors, there was a significant difference in uptake between astrocytic HGG and LGG. Although data not directly provided in this review but the presumed additional value of amino acid PET in presumed LGG may be estimated from the standard MRI approach where presumed LGG in fact have HGG focus in 21–41% [4, 14, 23, 43], while according to this review presumed LGG with high amino acid uptake in 28–94% is confirmed to be of a higher malignancy grade [4, 14, 18, 23, 43]. There is also a significant amount of HGG that is not detected by PET, with studies reporting negative PET in 12–19% of HGG [14, 15, 23]. This imperfect correlation between malignant focus in LGG was also recently using FET PET at time of suspected LGG progression [3].

### PET-guided biopsies towards areas of increased uptake

The second question touches on focal hot spots consisting of areas of increased uptake and their correlation with histopathological grading. MRI has suboptimal accuracy in determining glioma grade, especially when faced with non-enhancing gliomas with no or little edema [27]. Thus, the existence of hot spots is intriguing from a preoperative clinical situation to minimize sampling bias (i.e., sampling bias presumably less of a problem if a uniform high uptake is seen), but then hot spots must be confined to areas with highest malignancy grade. In most studies included in this review addressing this issue (4 of total 28), hot spots are not synonymous with HGG. Nevertheless, Kunz et al. [27] demonstrated a clear association between hot spots and higher grades of malignancy inside, compared to outside the hot spot, by analyzing the TAC within the tumor. While this particular study demonstrates that amino acid PET can potentially be of great value, their findings need to be validated by other research groups. Albert et al. [2] investigated the accuracy of tumor grading using tumor-to-brain ratio (TBR_max_) values at different time points after tracer injection, in order to establish the optimal time point for discriminating between LGG and HGG. Their findings showed that TBR_max_ values in early summation images are significantly better for tumor grading compared to standard static 20–40 min scans, proving that when dynamic 18F-FET is impossible to perform, early TBR_max_ assessment can be an alternative for PET-assisted tumor grading. Evaluating diagnostic yield compared to regular MRI was outside scope of this review, since we here specifically address the areas of increased uptake, and in most studies a substantial proportion of LGG (31–75%) have no increase in amino acid PET uptake [15, 18, 23]. Altogether, amino acid PET-guided biopsies seem clinical relevant and should be implemented to improve diagnostic accuracy in presumed LGG. If LGG are resected, targeted sampling may achieve similar results using intraoperative tools avoiding brain shift, and this is already demonstrated with the use of 5-ALA [48] and in the future methods like Raman spectroscopy may play a role [26].

### Independent prognostic information provided by preoperative PET imaging

Our third question evaluated the prognostic value of amino acid PET, after adjusting for established clinical prognostic factors. An important limitation in such studies is that the metabolically active part of the tumor is often resected later on. Further the relatively long survival of these patients, and multiple therapies underway, makes this a difficult task. Only a limited number of publications met the inclusion criteria and conflicting results were found. One included study adjusted for molecular factors, concluding that increased FET uptake seems to offer additional useful prognostic information not fully captured by the molecular tumor profile [44]. As shown in Table 3, the majority of studies did not perform adjustment by clinical prognostic factors, thus the additional prognostic information by PET in these primarily positive studies (with respect to PET and prognostication) [4, 5, 44] remains unknown. Ribom et al. [37] reported that the uptake of MET in the whole patient cohort was not a prognostic factor. When astrocytomas and oligodendrogliomas were examined separately, low MET uptake was prognostically favorable only in oligodendrogliomas. Suchorska et al. [42] evaluated dynamic 18F-FET uptake in gliomas and demonstrated that longer time-to-peak minimum (TTP_min_) correlated with longer overall survival in the subgroup of tumors with *IDH 1/2* mutation/1p19q-non-codel. The authors conclude that dynamic 18F-FET might provide additional prognostic information when stratifying astrocytoma patients into high- and low-risk groups. Currently, this should be focus to further research and additional prognostic information beyond molecular markers based on amino acid PET should not be used for clinical decision-making or provided directly to patients.

### Amino acid PET to predict molecular subgroups in LGG

Finally, we wanted to find out whether preoperative amino acid PET can be used to predict molecular subgroups among LGG, although we were aware of that most relevant PET literature were published prior to the 2016 WHO classification where molecular markers were integrated [11]. Of relevance, the IDH mutation and 1p19q codeletion do not only matter to classification but these markers also offer prognostic information [7]. The few included studies presented conflicting results with respect to the uptake of PET in different molecular classes, but they also varied in study design. Importantly, these studies used different amino acid tracers and different methods for detection of molecular markers (immunohistochemistry vs. sequencing), which might have contributed to the discrepancies*.* Nevertheless, based on these preliminary data, we are currently not able to identify molecular classes using current amino acid PET techniques. A recently published study by Verger et al. [46] concluded that static and dynamic 18-F-FET PET has a statistically significant role in discriminating between *IDH* mutated astrocytomas and *IDH* wild-type glioblastomas, although the method lacks value when discriminating between these two groups of gliomas and *IDH* mutated/1p19q codeleted oligodendrogliomas. Since most of the relevant literature is from the era before molecular markers, further research should be encouraged.

In summary, our review provides similar results as the recently published Response Assessment in Neuro-Oncology (RANO) recommendations for the clinical use of PET imaging in gliomas [1], bringing forward the problem with the significant overlap of tumor-to-brain ratio between tumors with different WHO grades as well as histological subtypes. Furthermore, this report concludes that it is favorable to evaluate dynamic 18F-FET PET data when differentiating between WHO grade II and WHO grade III/IV tumors. Similarly, we concur with the RANO recommendations that dynamic 18F-FET PET holds some promise for prognostication of astrocytomas. Our report differs from the RANO recommendations in terms of design, since we used the rigorous setup of a systematic review and that we focus solely on presumed LGG at baseline. In addition, we address if amino acid PET can be used for independent prognostic information or to predict molecular profiles, questions not readily addressed in the RANO report. Also, that another group that is not so attached to previous PET research confirms the major findings further strengthens the RANO recommendations being related to LGG.

### Strengths and limitations

A number of presumably relevant studies included in the literature search presented data only group-wise and were exclusively used as topic of discussion. Where we compare PET findings to subsequent histopathology, ideally the entire tumor volume should be assessed by histopathology to capture the “true” malignancy grade. Thus, the golden standard reported here in terms of histopathology is derived also from partial resections and biopsies and this may underestimate the true malignancy grade. Studies reporting on prognostic use of PET imaging many times lacked adjustment for clinical and molecular factors, leading to loss of important prognostic information. Finally, more advanced methods of analyzing data exist that could have been used instead of rating PET scans as “positive” versus “negative” as done in this review. As such, quantitative and multimodality data, frequently used in radiogenomics, are potential methods for analyzing PET images that are likely to refine results further [16, 17, 29]. Hence, data have been lost and this is a limitation when seeking an answer across many different studies with diverging procedures and assessments. Thus, since we reported positive PET scan, as interpreted by the authors themselves, we may have underestimated the best and overestimated the worst protocols. This marked heterogeneity in studies is also the reason why we did not attempt to perform a pooled analysis/meta-analysis and have provided only data from individual studies.

## Conclusions

Based on the available literature, different uptake values can be found between non-neoplastic lesions, LGG and HGG, but the overlap between tumor subtypes hampers clear separation. For detection of areas with higher malignancy, dynamic FET imaging seems superior to MRI and to other PET techniques. No clear benefit concerning the independent prognostic information from amino acid PET imaging was found, since studies were few and results were conflicting. Lastly, there is no current evidence that PET can be used to predict molecular subgroups of LGG.

*N/A* not available, *MET* 11C-methionine, *FET* 18F-fluoro-ethyl-tyrosine, *T/N ratio* tumor-to-normal uptake ratio, *TBR*_*max*_ tumor-to-background ratio max, *SUV/BG* standardized uptake values/background, *SUV*_*max*_ standardized uptake values max, *HS/cortex ratio* hot spot-to-cortex ratio, *WHO* World Health Organization, *R* retrospective, *P* prospective

## Electronic supplementary material

ESM 1 (DOCX 15 kb)
