# Supplementary material for: Amino acid tracers in PET imaging of diffuse low-grade gliomas: a systematic review of preoperative applications
Source: Acta Neurochir (Wien). 2018 May 24;160(7):1451–60. doi: 10.1007/s00701-018-3563-3 (PMC5995993; doi:10.1007/s00701-018-3563-3)
Supplement: Supplementary file 1 — (DOCX 15 kb) [file 701_2018_3563_MOESM1_ESM.docx]

**Supplementary material**

In order to develop this literature search strategy, the PIRO model was used (population, investigation, reference, outcome). Through this process, the following inclusion criteria was established:

P: Studies on adult (≥16 years) patients where PET imaging using radiolabeled amino-acid tracers have been performed in cases of suspected LGG (i.e. before any treatment is provided), and where later histopathology is available, published in the period from 1995 through 2016. In this context, we refer to LGGs as the diffuse gliomas represented by astrocytoma, oligodendroglioma and mixed WHO grade II.

I: LGG patients with preoperative (baseline) PET using amino-acid tracers.

R: Presumed low-grade glioma assessed by amino acid PET at baseline evaluated against later histopathology

O: In suspected low-grade gliomas later verified by histopathology, are PET helpful in differentiation non-tumoral diagnoses and high-grade gliomas from LGG?

In suspected LGG, is PET guided biopsies towards areas of increased uptake correlated to higher grade of malignancy?

In LGG, what is the prognostic information provided by preoperative PET imaging

after adjusting for other important prognostic variables?

Can preoperative PET imaging using amino-acid tracers predict molecular subgroups in LGG patients?
